# Scan Once, Analyse Many: Using Large Open-Access Neuroimaging Datasets to Understand the Brain

**DOI:** 10.1007/s12021-021-09519-6

**Published:** 2021-05-11

**Authors:** Christopher R. Madan

**Affiliations:** School of Psychology, University of Nottingham, Nottingham, NG7 2RD UK

**Keywords:** Brain imaging, Secondary data, Connectome, Sample size, Functional connectivity, fMRI, Naturalistic neuroimaging

## Abstract

We are now in a time of readily available brain imaging data. Not only are researchers now sharing data more than ever before, but additionally large-scale data collecting initiatives are underway with the vision that many future researchers will use the data for secondary analyses. Here I provide an overview of available datasets and some example use cases. Example use cases include examining individual differences, more robust findings, reproducibility–both in public input data and availability as a replication sample, and methods development. I further discuss a variety of considerations associated with using existing data and the opportunities associated with large datasets. Suggestions for further readings on general neuroimaging and topic-specific discussions are also provided.

## Introduction

It is a great time to be studying human brain development, aging, or differences between healthy individuals and variety of neurological patient conditions–massive amounts of already acquired and openly available MRI data exist. As long as you’re satisfied with existing data collection protocols (e.g., not developing a new MR sequence or cognitive task), the data you are looking for to test a novel measure of brain structure, connectivity, or task-related activation may be only a few clicks away. Data sharing has countless benefits, allowing for the ready assessment of new research questions, enhancing reproducability, providing initial ‘pilot’ data for new methods development, and reducing the costs associated with doing neuroimaging research (Mar et al. 2013; Poldrack and Gorgolewski 2014; Madan 2017; Milham et al. 2018). While there are some considerations needed related to over-fitting to specific datasets (Madan 2017), otherwise referred to as ‘dataset decay’ (Thompson et al. 2020), there is much we can learn from these existing datasets before we must go out and acquire new ones.

The availability of data sharing has greatly increased over the last few years, in no small part due to the development of the ‘FAIR guiding principles for scientific data management and stewardship’ (Wilkinson et al. 2016): Findability, Accessibility, Interoperability, and Reuse of digital assets. Adherence to the FAIR guidelines is further facilitated by consistent file organisation standards (i.e., Brain Imaging Data Structure; BIDS) (Gorgolewski et al. 2016). Other standards and guidelines are also advancing the methodological rigor of the field, such as the Committee on Best Practices in Data Analysis and Sharing (COBIDAS) MRI (Nichols et al. 2017), among other best-practice recommendations (Eglen et al. 2017; Shenkin et al. 2017). Typical MRI studies can be readily shared using platforms including OpenNeuro (Poldrack and Gorgolewski 2017), allowing for further analyses of the data by other research groups, as well as assessments of analysis reproducability, though large-scale projects may require more dedicated infrastructure (discussed later).

**Fig. 1:**
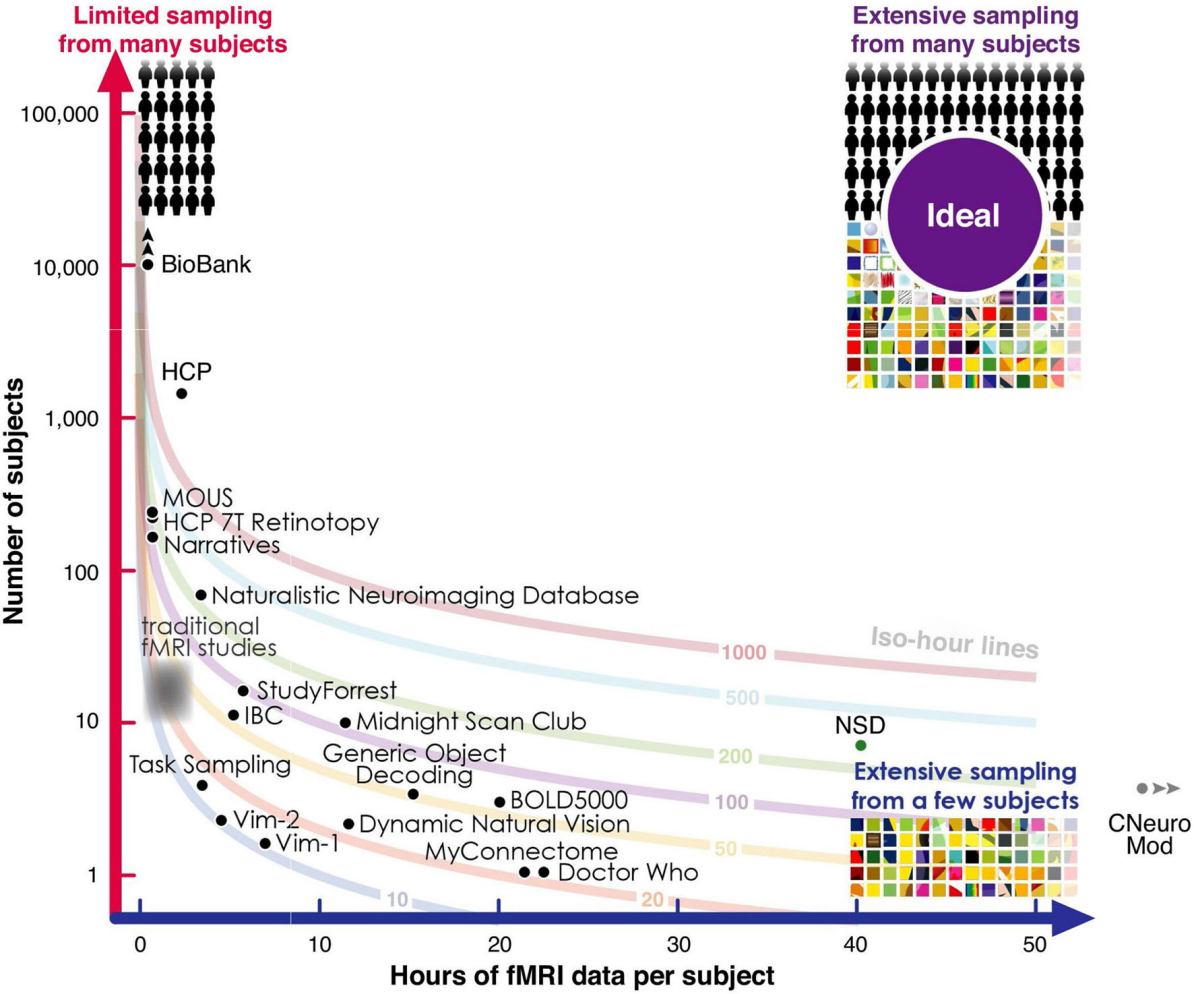
Trade-offs between number of participants and amount of data per participant. Note that some datasets have increased in size since the generation of this figure (e.g., IBC has more data per participant now); some datasets are not featured in the current review, e.g., VIM-1. Reprinted from Naselaris et al. (2021)

Here I will focus on the availability of large-scale neuroimaging datasets that help us move beyond the statistical power issues that are still typical within the field (Button et al. 2013; Zuo et al. 2019) and more towards furthering our understanding of the brain. This shift towards large-scale datasets can also be important for individual analyses, as these large datasets provide a more meaningful opportunity to shift from group averaging to comparing the statistics of individual participants (Dubois and Adolphs 2016), bolstered by multimodal acquisitions and highly sampled individuals, providing richer insights into individual brains and their relative differences. Naselaris et al. (2021) provides an insightful discussion for considering trade-offs between sampling more individuals as compared to more experimental data from a few individuals (e.g., considering a fixed amount of total scan time), as summarised in Fig. 1.

The magnitude of considerations needed when designing a large-scale dataset cannot be understated. For instance, a myriad of topics related to the design of the Human Connectome Project (HCP) were discussed in a special issue of *NeuroImage* in 2013 (volume 80) (e.g., Van Essen et al. 2013) and similar was done for the Adolescent Brain Cognitive Development (ABCD) consortium in *Developmental Cognitive Neuroscience* in 2018 (volume 32) (e.g., Casey et al.^2018^). While it is relatively easy to use these datasets in your own research, I think it is also important to be aware of the considerations that were made when when they were developed. For instance with the ABCD study, it may be useful to further consider how the physical and mental health assessments were chosen (Barch et al. 2018) as well as the ethical considerations that were made, since the study involves the multi-site recruitment of children and adolescents (Clark et al. 2018). For examples of the considerations that may need to be made when collecting data with a clinical sample, see Ye et al. (2019). Its additionally important to evaluate how other data collection considerations, such as the MR sequences used, may influence analyses, e.g., multiband sequences improve temporal resolution, but can also introduce slice-leakage artifacts (Todd et al. 2016; Risk et al. 2018; McNabb et al. 2020). (For a critical discussion, see Longo and Drazen (2016).)

## Large Open-Access Neuroimaging Datasets

Over the last two decades, but particularly in recent years, many large open-access neuroimaging datasets have become available (e.g., Marcus et al. 2007; Jack et al. 2008; Van Essen et al. 2013; Hanke et al. 2014; Zuo et al. 2014; Poldrack et al. 2015; Alexander et al. 2017; Taylor et al. 2017; Harms et al. 2018; Casey et al.^2018^; Milham et al. 2020; Pinho et al. 2020; Nastase et al. 2020). In Table 1, I provide an overview of many of these, spanning multimodal investigations of young adults, lifespan studies of development and/or aging, highly sampled individuals, patient samples, as well as datasets of non-human neuroimaging. Here I have focused on relatively large, novel, or otherwise popular datasets. For instance, OpenNeuro (formerly OpenfMRI) has recently surpassed 500 public datasets, however, most of these are ‘conventional’ in scale (e.g., < 40 participants, single session, sparse additional non-imaging data) and, as such, are not included in the table. These are, of course, still very useful, but their smaller scale makes their applications more limited than the datasets emphasised in this overview. Several schizophrenia datasets are included in the table that are part of SchizConnect (Wang et al. 2016), however, these are also listed separately since they are in federated databases and are otherwise disparate and heterogeneous.

**Table 1:** Overview of large open-access neuroimaging datasets

				Accessibility	
Short name	Full name	N	Comments	Score	Reference
	*young adults*				
HCP	Human Connectome Project (Young Adult)	1200	ages 22-35, multimodal, genetics, task fMRI, behaviour, 7 T, partial test-retest (2, N = 45)	2,3,4	Van Essen et al. (2013); Glasser et al. (2016)
GSP	Brain Genomics Superstruct Project	1570	ages 18-35, resting state, behaviour, partial test-retest (2, N = 69)	2,3	Holmes et al. (2015)
AOMIC	Amsterdam Open MRI Collection	1370	ages 19-26, multimodal, task fMRI, behaviour	1	Snoek et al. (2021)
CoRR	Consortium for Reliability and Reproducibility	1629	resting state, test-retest (2+), multi-site (18), global	1	Zuo et al. (2014)
7T-TRT	–	22	ages 21-30, multimodal, resting state, test-retest, behaviour, subset of CoRR (MPG1)	1	Gorgolewski et al. (2015)
CCBD	Center for Cognition and Brain Disorders	30	ages 20-30, resting state, test-retest (10), subset of CoRR (HNU1)	1	Chen et al. (2015)
–	Test-Retest Reliability of Brain Volume Measurements	3	ages 26-31, test-retest (20)	1	Maclaren et al. (2014)
SLIM	Southwest University Longitudinal Imaging Multimodal	595	ages 17-27, resting state, test-retest, China	1	Liu et al. (2017)
NARPS	Neuroimaging Analysis Replication and Prediction Study	119	ages 18-37, task fMRI	1	Botvinik-Nezer et al. (2019) and Botvinik-Nezer et al. (2020)
	*lifespan and aging*				
NKI-RS/eNKI	Enhanced Nathan Kline Institute – Rockland Sample	> 1000	on-going (current: 1334), ages 6-85, community sample, multimodal, task fMRI, behaviour	1,4	Nooner et al. (2012)
IXI	Information eXtraction from Images	581	ages 20-86, multi-site (3), multimodal	1	Keihaninejad et al. (2009)
Kirby-21/MMRR	Multi-Modal MRI Reproducibility Resource	21	ages 22-61, multimodal, test-retest (2)	1	Landman et al. (2011)
DLBS	Dallas Life Brain Study	315	ages 20-89, multimodal, task fMRI, behaviour, genetics	1,4	Kennedy et al. (2015)
ICBM	International Consortium for Brain Mapping	853	ages 18-80, multi-site (3)	3	Mazziotta et al. (2009)
SALD	Southwest University Adult Lifespan Dataset	494	ages 19-80, resting state, China	1	Wei et al. (2018)
LEMON	MPI Leipzig Study for Mind-Body-Emotion Interactions	228	ages 20-35 and 59-77, multimodal, resting state, behaviour	1	Babayan et al. (2019)
N&C	MPI Leipzig Neuroanatomy & Connectivity	321	ages 20-75, multimodal, resting state, behaviour	1	Mendes et al. (2019)
CC-359	Calgary-Campinas-359	359	ages 29-80, multi-site (6)	1	Souza et al. (2018)
CHBMP	Cuban Human Brain Mapping Project	203	ages 18-68, multimodal, behaviour	3	Valdes-Sosa et al. (2021)
YaleLowres	Yale Low-Resolution Controls	100	ages 18-66, resting state	1	Scheinost et al. (2015)
YaleHires	Yale High-Resolution Controls	120	ages 18-58, resting state	1	Finn et al. (2015)
YaleTRT	Yale Test-Retest	12	ages 27-56, resting state, test-retest (4)	1	Noble et al. (2017)
MOUS	Mother Of Unification Studies	204	ages 18-33, multimodal, task fMRI	2	Schoffelen et al. (2019)
NNdb	Naturalistic Neuroimaging Database	86	ages 18-58, movie watching, behaviour	1	(Aliko et al. 2020)
Narratives	–	345	ages 18-53, story listening	1	Nastase et al. (2020)
HCP-A	Lifespan Human Connectome Project in Aging	> 1200	on-going (current: 725), ages 36-100, multi-site (4), multimodal, task fMRI, behaviour	4	Bookheimer et al. (2019)
OMEGA	Open MEG Archive	220	ages 21-75, multimodal, task fMRI	3	Niso et al. (2016)
CamCAN	Cambridge Center for Ageing and Neuroscience	700	ages 18-87, community sample, multimodal, task fMRI, movie watching, genetics	3	Taylor et al. (2017); Henson et al. (2020)
BLSA	Baltimore Longitudinal Study of Aging	> 1000	on-going (current: 889; long.), ages 50-90, community sample, longitudinal, task fMRI, behaviour, genetics	4	Ferrucci (2008) and Armstrong et al. (2019)
BASE-II	Berlin Aging Study II	2,200	on-going (long.), ages 20-35 and 60-80, multimodal, behaviour, genetics	4	Bertram et al. (2014)
UKBB	UK Biobank Imaging study	100,000	on-going (current: 43k; long.), ages 35-80, community sample, longitudinal, multimodal, task fMRI, behaviour	4	Miller et al. (2016) and Littlejohns et al. (2020)
LBC1936	Lothian Birth Cohort 1936	1091	on-going (long.), ages 70-82, longitudinal, behaviour	4	Taylor et al. (2018)
Allen	Allen Human Brain Atlas	8	post-mortem, ages 24-57, multimodal, histology	1	Hawrylycz et al. (2012)
BigBrain	–	1	post-mortem, age 65, histology	1	Amunts et al. (2013)
	*development*				
–	Theory of Mind development	155	ages 3-12 and 18-39, movie watching, behaviour	1	Richardson et al. (2018)
dHCP	Developing Human Connectome Project	> 1000	on-going (current: 538), ages 20-45 weeks, multimodal, resting state	2	Hughes et al. (2017), Makropoulos et al. (2018), and Fitzgibbon et al. (2020)
ABCD	Adolescent Brain Cognitive Development study	> 10,000	on-going (current: 11,878; long.), ages 9-10 until 20, longitudinal, multi-site (21), multimodal, task fMRI	4	Casey et al. (2018)
HCP-D	Lifespan Human Connectome Project in Development	> 1350	on-going (current: 655), ages 5-21, multimodal, multi-site (4), task fMRI	4	Somerville et al. (2018)
PING	Pediatric Imaging, Neurocognition, and Genetics	1493	ages 3-20, multi-site (10), multimodal, resting state, genetics	3	Jernigan et al. (2016)
PNC	Philadelphia Neurodevelopmental Cohort	1445	ages 8-21, multimodal, task fMRI, genetics	4	Satterthwaite et al. (2016)
HBN	Healthy Brain Network	> 10,000	on-going (current: 3625), ages 5-21, multimodal, task fMRI, movie watching, behaviour	4	Alexander et al. (2017)
PedsMRI	NIH Pediatric MRI Data Repository	500	ages birth-4, longitudinal, multimodal, multi-site (6), behaviour	4	Brain Development Cooperative Group and Evans (2006)
IBIS	Infant Brain Imaging Study	> 900	on-going (current: 503; long.), ages 6-24 months, longitudinal, multimodal, multi-site (5)	4	Hazlett et al. (2012) and Cárdenas-de-la-Parra et al. (2021)
Dev-CoG	Developmental Chronnecto-Genomics	> 200	on-going (no data release yet), ages 9-14, multi-site (2), longitudinal, multimodal, task fMRI, behaviour, genetics	4	Stephen et al. (2021)
	*highly sampled individuals*				
MyConnectome	–	1	aged 45, repeated scans (84, spanning 18 months), multimodal, task fMRI	1	Poldrack et al. (2015) and Poldrack (2020)
MSC	Midnight Scan Club	10	repeated scans, multimodal, task fMRI, behaviour	1	Gordon et al. (2017)
Kirby Weekly	Single-subject Resting state fMRI Reproducibility Resource	1	aged 40, repeated scans (158, spanning 3.5 years), resting state	1	Choe et al. (2015)
SIMON	Single Individual volunteering for Multiple Observations across Networks	1	aged 29-46, repeated scans (73, spanning 15 years), mutli-site (36), multimodal, task fMRI	1	Duchesne et al. (2019)
IBC	Individual Brain Charting	12	on-going (long.), ages 26-41, repeated scans, task fMRI, movie watching	1	Pinho et al. (2018) and Pinho et al. (2020)
Studyforrest	–	20	multimodal, task fMRI, movie watching	1	Hanke et al. (2014)
BOLD5000	Brain, Object, Landscape Dataset	4	ages 24-27, repeated scans, multimodal, task fMRI	1	Chang et al. (2019)
NSD	Natural Scenes Dataset	8	ages 19-32, repeated scans, multimodal, task fMRI	3	Naselaris et al. (2021) and Allen et al. (2021)
Sherlock	–	16	movie watching, behaviour	1	Chen et al. (2017)
T1 250 *μ* m	–	1	T1 scans at various resolutions (up to 250 *μ* m)	1	Lusebrink et al. (2017)
MASSIVE	Multiple Acquisitions for Standardization of Structural Imaging Validation and Evaluation	1	8000 dMRI volumes with various parameters, along with T1 and T2 scans	1	Froeling et al. (2017)
Doctor Who	–	1	age 27, task fMRI, movie watching		Seeliger et al. (2019)
C-NeuroMod	Courtois Project on Neuronal Modelling	6	on-going (repeated scans), repeated scans, multimodal, task fMRI, movie watching	4	Bellec and Boyle (2019)
	*dementia related*				
OASIS-1	Open Access Series of Imaging Studies 1: Cross-sectional MRI Data in Young, Middle Aged, Nondemented and Demented Older Adults	416	ages 18-96, repeated scans (4)	1	Marcus et al. (2007)
OASIS-2	Longitudinal MRI Data in Nondemented and Demented Older Adults	150	ages 60-96, repeated scans (4), longitudinal	1	Marcus et al. (2010)
OASIS-3	Longitudinal Neuroimaging, Clinical, and Cognitive Dataset for Normal Aging and Alzheimer’s Disease	1098	ages 42-95, longitudinal, multimodal, resting state, genetics	3	LaMontagne et al. (2019)
ADNI	Alzheimer’s Disease Neuroimaging Initiative	> 3500	on-going (current: 3228; long.), ages 55-90, longitudinal, multimodal, resting state, multi-site (57), genetics	3	Jack et al. (2008)
AIBL	Australian Imaging, Biomarker & Lifestyle	863	ages 55-96, longitudinal, multimodal, genetics, borrowed methods from ADNI	3	Ellis et al. (2009)
PREVENT-AD	Pre-symptomatic Evaluation of Experimental or Novel Treatments for Alzheimer Disease	425	ages 55-85, test-retest, longitudinal, multimodal, task fMRI, behaviour, genetics	3	Orban et al. (2015), Breitner et al. (2016), and Tremblay-Mercier et al. (2020)
HABS	Harvard Aging Brain Study	290	on-going (long.), ages 62-90, longitudinal, multimodal, task fMRI, behaviour, genetics	4	Dagley et al. (2017)
MAP	Rush Memory and Aging Project	> 2100	on-going (current: 936 with MRI; long.), ages 65+, longitudinal, behaviour, genetics, histology	4	Bennett et al. (2012) and Lamar et al. (2020)
COMPASS-ND	Comprehensive Assessment of Neurodegeneration and Dementia	2300	on-going (current: 1132), ages 50-90, multi-site (30), longitudinal, behaviour	4	Chertkow et al. (2019)
	*other clinical samples*				
ABIDE	Autism Brain Imaging Data Exchange	1112	autism, ages 7-64, multi-site (17), resting state	1	Di Martino et al. (2014)
ABIDE2	Autism Brain Imaging Data Exchange II	2156	autism, ages 5-64, mutli-site (19), multimodal, resting state	1	Martino et al. (2017)
ADHD-200	ADHD-200 Global Competition	973	ADHD, ages 7-27, multi-site (8), resting state	1	ADHD-200 Consortium (2012); Fair et al. (2013) and Bellec et al. (2017)
SchizConnect	–	1400	schizophrenia, ages 14-67, multimodal, multi-site, task fMRI	3	Wang et al. (2016)
COBRE	Centers of Biomedical Research Excellence	146	schizophrenia, ages 18-65, resting state, behaviour, subset of SchizConnect	1	Çetin et al. (2014) and Aine et al. (2017)
MCIC	MIND Clinical Imaging Consortium	331	schizophrenia, ages 18-60, multi-site (4), multimodal, task fMRI, behaviour, genetics, subset of SchizConnect	3,4	Gollub et al. (2013)
NUSDAST	Northwestern University Schizophrenia Data and Software Tool	451	schizophrenia, ages 17-67, multimodal, behaviour, genetics, subset of SchizConnect	3	Wang et al. (2013)
CANDI	Child and Adolescent NeuroDevelopment Initiative	103	schizophrenia, bipolar disorder, ages 4-17	1	Frazier et al. (2008)
LA5c	UCLA Consortium for Neuropsychiatric Phenomics	273	schizophrenia, bipolar disorder, ADHD, ages 21-50, multimodal, task fMRI	1	Poldrack et al. (2016)
PPMI	Parkinson Progression Marker Initiative	600	Parkinson’s disease, ages 30-87, multimodal, multi-site (21)	3	Marek et al. (2011)
QPN	Québec Parkinson Network	> 2000	Parkinson’s disease, on-going (current:1070), ages 33-94, multimodal	4	Gan-Or et al. (2020)
TRACK-HD	Track Huntington’s Disease	366	Huntington’s disease, ages 18-65, multi-site (4), multimodal, behaviour, genetics	4	Tabrizi et al. (2009)
SRPBS-MD	Strategic Research Program for Brain Sciences - Multi-Disorder	805	several patient groups, mutli-site (9)	3	Yamashita et al. (2019)
PAIN	Pain and Interoception Imaging Network	973	pain-related conditions, multi-site, resting state, behaviour	4	Labus et al. (2016)
MNI Open iEEG	–	106	intracranial EEG	3	Frauscher et al. (2018)
RAM	Restoring Active Memory	251	intracranial EEG	3	Weidemann et al. (2019)
	*non-human*				
PRIME	PRIMatE Data Exchange	> 100	primate, on-going (current: 227), multi-site, task fMRI	2,4	Milham et al. (2020)
MNDD	UNC-Wisconsin Rhesus Macaque Neurodevelopment Database	34	macaque, longitudinal, multimodal	1	Young et al. (2017)
–	Awake Rat rsfMRI Database	90	rat, resting-state	1	Liu et al. (2020)
–	Mouse rest multicentre	255	mouse, resting-state, multi-site (17)	1	Grandjean et al. (2020)

Projects that are “on-going” have the target **N** listed, though the current available sample size (as of February 2021) or if the on-going nature is related to the collection of subsequent longitudinal timepoints. Numbers after “multi-site” indicate the number of sites. “resting state” indicates that resting-state fMRI data is available (but not task), “task fMRI” indicates that task, and often resting-state, fMRI data are available; “multimodal” indicates that other imaging modalities are available beyond T1-weighted and fMRI data; “behaviour” indicates that a substantial amount of non-fMRI behavioural measures; “genetics” indicates that at least some genetics data was also acquired and is shared. Sample sizes for the dementia and other clinical samples include all individuals (i.e., including matched healthy controls). See main text for a detailed description of the accessibility score

While these datasets are all considered open-access, there is variation in how easy it is to get access to the data. Based on the level of effort required to access the data, I have here coded them each with an “accessibility score” on a 4-point scale: (1) minimal data use agreement required, automatic approval (e.g., IXI, OASIS1, ABIDE, COBRE); (2) some study-specific terms in agreement–to be read carefully, still automatic approval (e.g., HCP, GSP); (3) applications manually approved, often requiring a brief application including a study plan or analysis proposal (e.g., ADNI, CamCAN); (4) more extensive data-use application, requiring institutional support and/or lawyer involvement (e.g., ABCD, HBN). Some datasets have been coded with multiple scores, in cases where some data is shared more readily, but additional variables are provided under restricted terms. As an example, the Human Connectome Project (HCP) is coded as ‘2,3,4.’ While the HCP data is readily shared, it does involve some specific terms, such as not using participant IDs publicly, such as in publications (e.g., including in figures). Additional restricted data (e.g., medical family history) are available under formal application; moreover, genetic data is overseen through NIH dbGaP (‘the Database of Genotypes and Phenotypes’) and requires institutional supporting paperwork and approval.

Consideration is needed when combining data from multiple sites or datasets. It is well-established that there are site effects in MRI in a variety of derived measures. Hagiwara et al. (2020) provide a useful overview of statistics for comparing related measurements, as well as of common imaging-related of variance (e.g., temperature, field nonuniformity, and field strength). Data harmonisation can be attempted at either the initial 3D volume (e.g., signal-to-noise ratio) or specific derived measures (e.g., mean and variance of mean cortical thickness–for instance, using normalised residuals, subsequently combined across sites using site-specific scaling factors) with the goal of matching dataset descriptive statistics. The specific goals of harmonising are important to evaluate. For instance, two sites may exhibit age-related differences in mean cortical thickness, but have different within-site average estimates and age-related slopes. This could be due to site-specific differences in estimated tissue contrast and thus carry forward to the subsequent tissue segmentation and surface reconstruction. Estimates can be adjusted using within-site normalisation along with a linear combination of the site-specific scaling factors. More complex approaches for unseen data are being developed, with between-site harmonisation serving as an active field of methods development, particularly as the availability of open-access datasets continues to increase.

When providing an overview of these large-scale datasets, in addition to crediting the data generators themselves (Pierce et al. 2019), it is also important to acknowledge the software infrastructure that supports them (Ince et al. 2012; Barba et al. 2019). Many of these projects rely on software packages such as the Extensible Neuroimaging Archive Toolkit (XNAT) (Marcus et al. 2007; Herrick et al. 2016)–which was adapted into ConnectomeDB for the HCP (Marcus et al. 2011; Hodge et al. 2016), Collaborative Informatics Neuroimaging Suite (COINS) (Scott et al. 2011; Landis et al. 2016), Longitudinal Online Research and Imaging System (LORIS) (Das et al. 2012), or other online infrastructure such as the Neuroimaging Informatics Tools and Resources Clearinghouse (NITRC) (Kennedy et al. 2016), the International Neuroimaging Datasharing Initiative (INDI) (Mennes et al. 2013), the Laboratory of Neuro Imaging (LONI) (Crawford et al. 2016), and OpenNeuro (formerly OpenfMRI) (Poldrack et al. 2013; Poldrack and Gorgolewski 2017). These generally are ‘behind-the-scenes,’ but the data sharing and future analyses from these datasets is as dependent on these software packages as they are on the MRI scanners themselves. While small-scale, within-lab projects can proceed without these packages, they become integral when MRI data is being shared with large groups of users and metadata is linked closely to the individual MRI volumes. Moreover, shared data is often evaluated with some quality control as an initial preprocessing when shared, such as MRIQC (Esteban et al. 2017), fMRIprep (Esteban et al. 2019), PreQual (Cai et al. 2020), or Mindcontrol (Keshavan et al. 2018). Initiatives such as Open Brain Consent (Bannier et al. 2020) are also critical in making neuroimaging data more readily shared (also see Brakewood and Poldrack 2013; Shenkin et al. 2017; White et al. 2020). As a field, we also need to consider long-term data preservation; some previous repositories have become no longer accessible (e.g., fMRI Data Center [fMRIDC] and Biomedical Informatics Research Network [BIRN]) (Horn et al. 2001; Horn and Gazzaniga 2013; Helmer et al. 2011; Hunt 2019).

The overall approach here is ‘scan once, analyse many’ (adapted from the adage ‘write once, read many’ used to describe permanent data storage devices), and as such the benefits of streamlining of the data access process, such as due to these outlined software packages and sharing initiatives, benefits hundreds of ‘secondary analysis’ research groups. For instance, beyond sharing the primary data as a ‘data generator,’ openly sharing quality control (QC), preprocessed data, and data annotations (e.g., manual segmentations) saves others from repeating those efforts.

## Example Use Cases

Many innovative studies have already been conducted solely using data from one or more of the datasets outlined in Table 1. Here I provide some examples of this work, to help inspire and demonstrate what can be done using these large-scale open-access neuroimaging datasets. Four general categories of such studies, which particularly benefit from the opportunities created by large datasets, include studies of individual-difference analyses, robust findings, reproducability, and novel methodological findings that may not have been feasible to assess without using existing data. For more exhaustive lists of use cases for these databases, check their respective websites as many of them maintain lists of publications that have relied on their data.

### Individual Differences

Many of the findings presented in these example use cases could not have been established in ‘regular’ studies with conventional sample sizes. In particular, studies of individual differences require even larger sample sizes than for within-subject or group differences, and thus particularly benefit from the large-scale of these datasets. Functional connectivity analyses based on network graph-theory methods have become a prominent approach to examine individual differences, and this has been largely reliant on the availability of high-quality fMRI data from large samples (Yeo et al. 2014; Finn et al. 2015; Gratton et al. 2018; Greene et al. 2018; Greene et al. 2020; Seitzman et al. 2019; Cui et al. 2020; Salehi et al. 2020). Spronk et al. (2021) examined functional connectivity in several psychiatric conditions using open datasets (ADHD-200, ABIDE, COBRE), only finding subtle differences in network structure relative to healthy individuals.

The consideration of sex differences in neuroscience is being increasingly discussed, i.e., ‘sex as a biological variable (SABV)’ (Bale and Epperson 2016; Podcasy and Epperson 2016). This is a particularly fitting use of large, open-access neuroimaging datasets, as the inclusion of sex as a factor is unlikely to be an issue given the large sample sizes. Forde et al. (2020) examined sex differences in brain structure across the lifespan, using PNC, HCP, and OASIS-3 datasets (N = 3069). They observed an interaction, where males had less within-region variability in early years, but more variability in later years. This result extended previous work that had looked at narrower age ranges, such as Wierenga et al. (2018) with PING, more coarse brain size measures (e.g., van der Linden et al. 2017, with HCP), or more specific regions (e.g., van Eijk et al. 2020, with the hippocampus). Other studies have used these datasets to examine sex-related differences in brain activity or functional connectivity (e.g., Scheinost et al. 2015; Dumais et al. 2018; Dhamala et al. 2020; Li et al. 2020). A handful of studies have also examined brain structure or function differences in relation to personality traits (Riccelli et al. 2017; Gray et al. 2018; Nostro et al. 2018; Owens et al. 2019; Sripada et al. 1900). Some results indicate that personality should be examined separately for each sex (Nostro et al. 2018); many results appear replicable, but effects are relatively weak.

Examining age-related differences in brain structure and function has become a prominent topic in studies that use large open-access datasets. Some of this work is described below, in the methods development section, as it was associated with the development of novel methods. Additionally, using the movie watching data from CamCAN, Geerligs and Campbell (2018) examined differences in inter-participant synchrony and found age-related differences in how shared functional networks were activated, corresponding to processing of naturalistic experiences. In a subsequent study, Reagh et al. (2020) examined the same movie-watching fMRI data and observed increases in posterior (but not anterior) hippocampal activity in relation to event boundaries, but also that these increases were attenuated by aging (also see Ben-Yakov and Henson 2018).

**Fig. 2:**
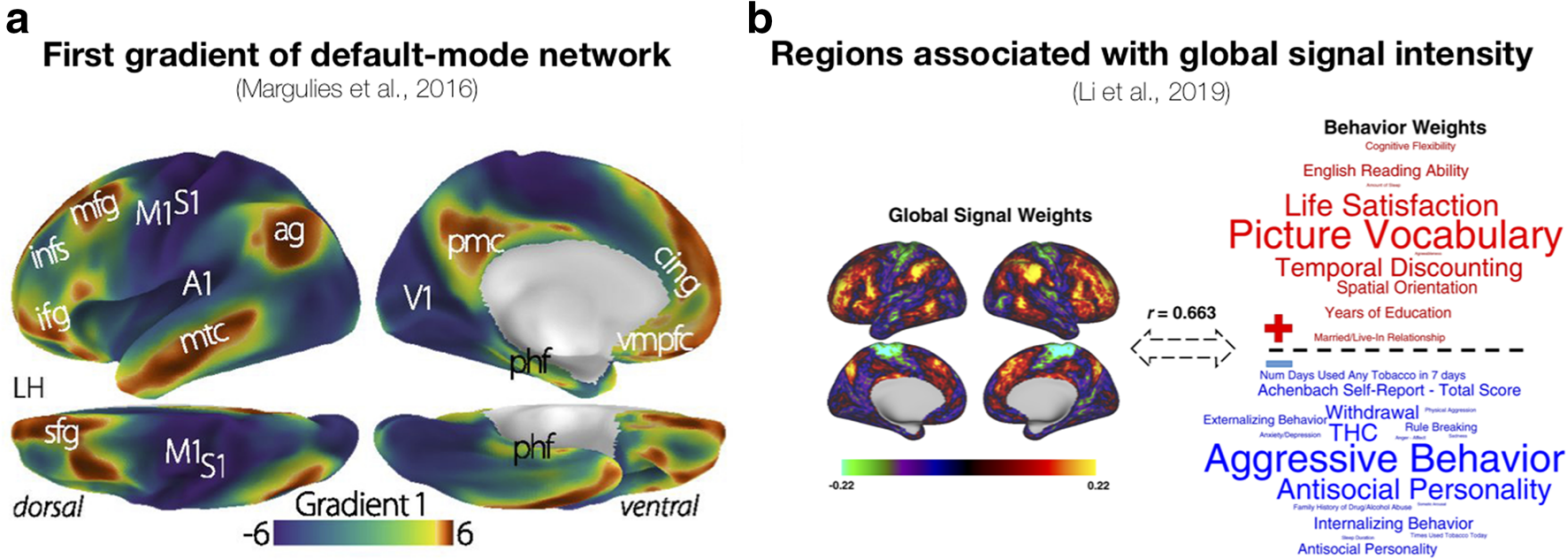
Robust resting-state activity patterns. **a** First gradient within the default-mode network, adapted from Margulies et al. (2016). **b** Regions associated with global signal intensity, adapted from Li et al. (2019)

**Fig. 3:**
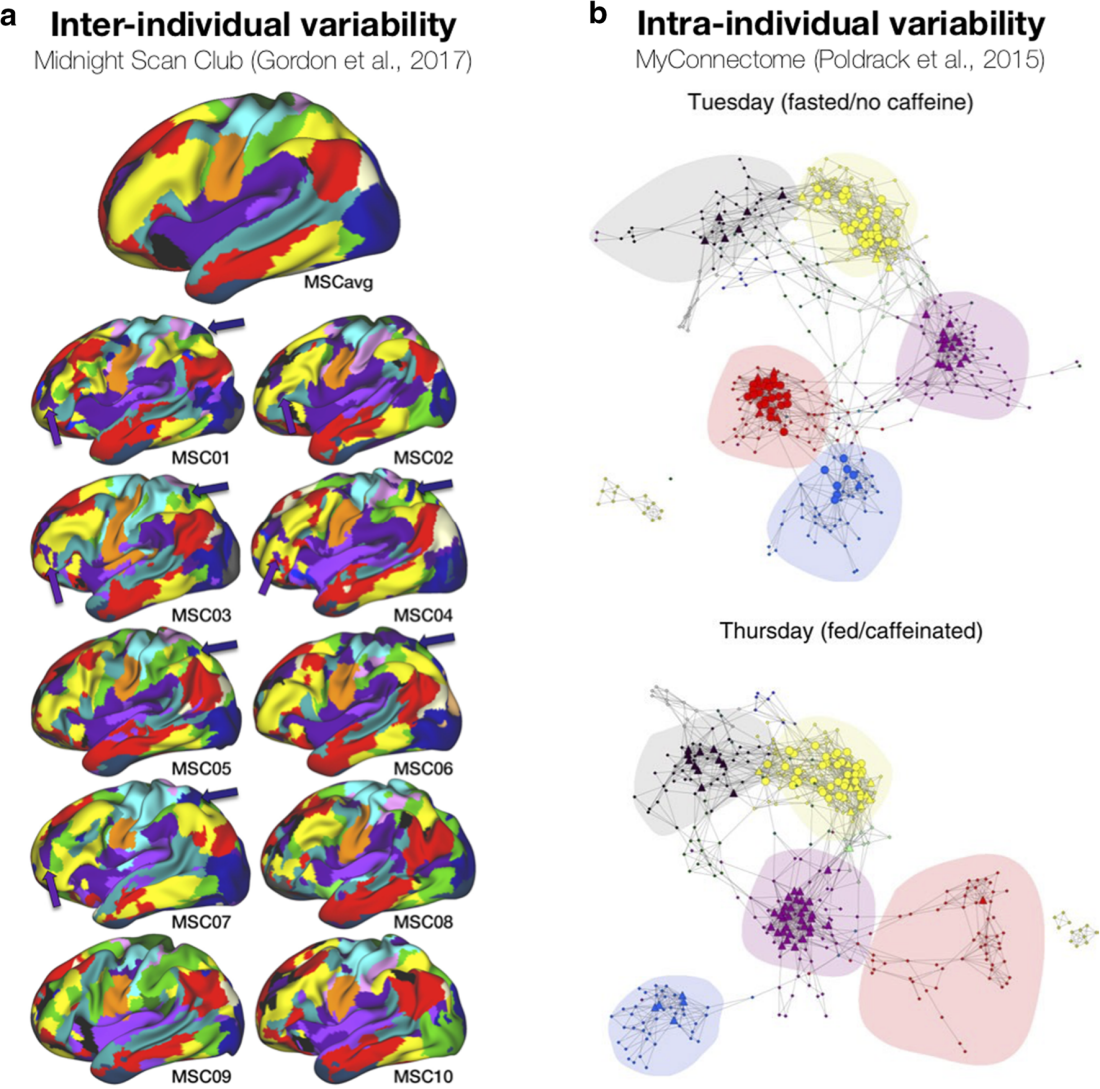
Inter- and intra-individual differences in functional connectivity from highly-sampled individuals. **a** Inter-individual variability across 10 individuals, reprinted from Gordon et al. (2017). **b** Intra-individual variability (related to fasting/caffeination), reprinted from Poldrack et al. (2015). Distinct colours denote each functional network. Arrows highlight specific regions of inter-individual variability

Several studies have been investigating individual differences in global fMRI signal (and the potential utility and limitations of global signal regression). This includes examining differences in relation to scan acquisitions and psychiatric conditions (Power et al. 2017), as well as behavioural features (Li et al. 2019), as shown in Fig. 2b (also see Smith et al. 2015). Others have examined the reproducability of individual differences and how data collection is influenced by multi-site factors, such as in the ABIDE (Abraham et al. 2017) and ABCD (Marek et al. 2019) studies. Within highly-sampled individuals, detailed network analyses can be conducted for each participant (Gordon et al. 2017) (Fig. 3a) and intra-individual differences can also be examined, such as the influence of caffeine on functional connectivity (Poldrack et al. 2015) (Fig. 3b) and BOLD signal variability (Yang et al. 2018).

In addition to using individual difference measures as continuous measures, some studies have used large-scale datasets to characterise potential subtypes within patient samples. Using different analytical approaches, Dong et al. (2017) and Zhang et al. (2016) demonstrated heterogeneity and subtypes in atrophy patterns due to Alzheimer’s disease using data from ADNI. Guo et al. (2020) also examined subtypes in the ADNI dataset, but instead focused on those with mild cognitive impairment. Furthermore, large open-access datasets have also been used to develop subtyping methods for autism (Easson et al. 2019; Tang et al. 2020) and schizophrenia (Castro-de-Araujo et al. 2020).

Other studies have examined differences in structure. For instance, Holmes et al. (2016) used the GSP dataset and examined relationships between cortical structure and different individual difference measures of cognitive control (e.g., sensation seeking, impulsivity, and substance use [alcohol, caffeine, and cigarettes]). Cao et al. (2017) examined gyrification trajectories across the lifespan, from 4 to 83, and in relation to several psychiatric conditions (major depression disorder, bipolar disorder, and schizophrenia), by combining data from a within-lab sample with the NKI and COBRE datasets.

**Fig. 4:**
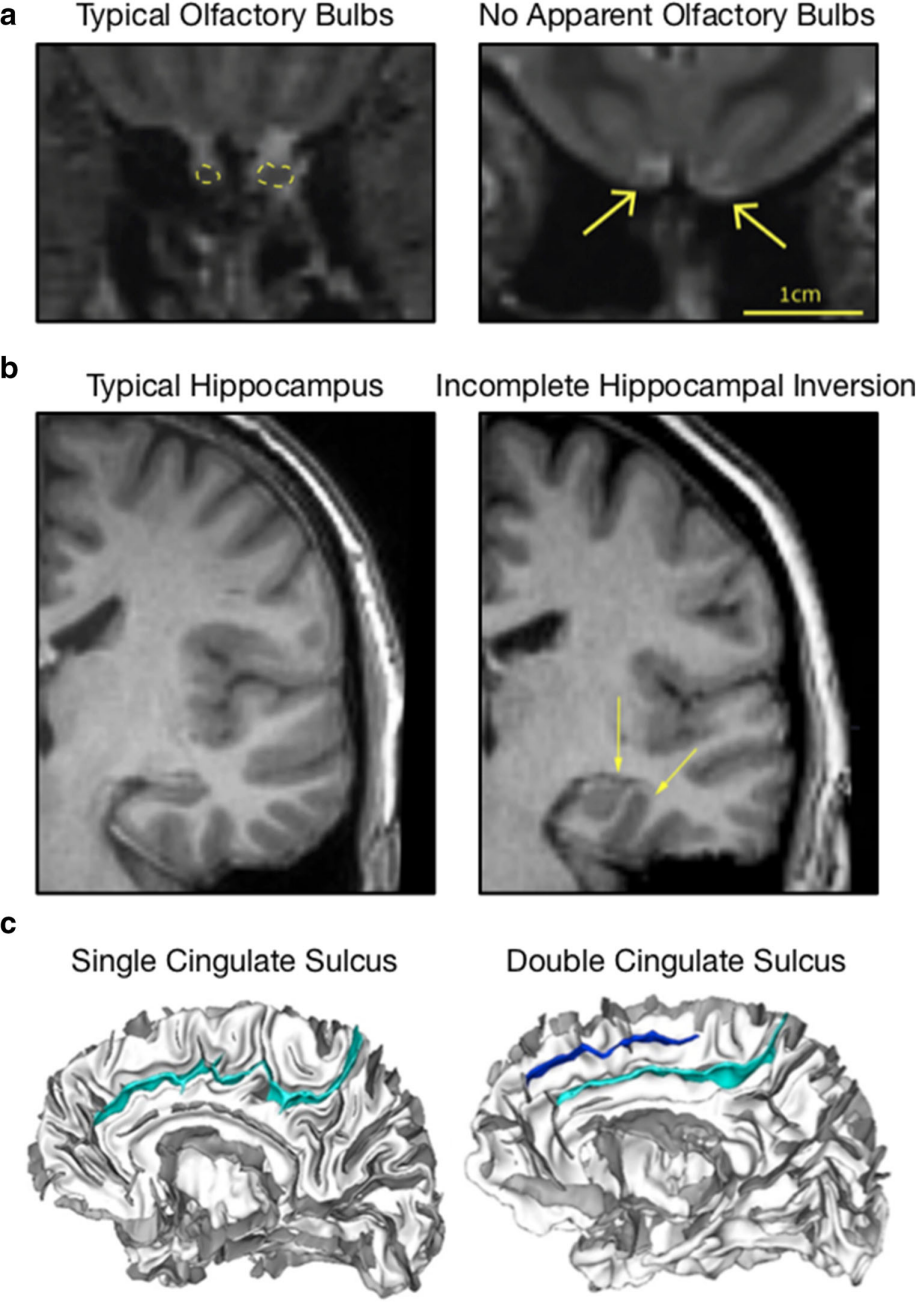
Examples of infrequent morphological features examined in large datasets. **a** Typical olfactory bulbs and no apparent bulbs in monozygotic twins, shown on a T2-weighted coronal image, adapted from Weiss et al. (2020). **b** Typical hippocampus and incomplete hippocampal inversion, shown on a T1-weighted coronal image, adapted from Caciagli et al. (2019). **c** Single and double cingulate sulcus, shown on the medial view of a reconstructed cortical surface, adapted from Cachia et al. (2016).

A narrow, but particularly beneficial use of these large datasets is to examine the frequency of infrequent brain morphological features. In conducting a conventional study, Weiss et al. (2020) identified two participants with no apparent olfactory bulbs, despite no impairments in olfactory performance. To examine the prevalence in the general population, the researchers examined the structural MRIs of 1113 participants from the HCP study. Three participants were identified–all had monozygotic twins that had visible olfactory bulbs. MRIs from one set of twins is shown in Fig. 4a. Moreover, the twins without apparent olfaction bulbs had *higher* olfaction scores than their twins with visible bulbs. A fourth participant was also identified, though the MRI was sufficiently blurry that it is difficult to be confident if the olfactory bulb is present. All identified individuals without apparent olfactory bulbs were women, occurring in 0.6% of women, with an increased likelihood in left-handed women (4.3%). Another morphological variation examined in large datasets is the incomplete hippocampal inversion, sometimes referred to a hippocampal malrotation, as shown in Fig. 4b. This can be identified by the diameter and curvature of the hippocampus, as well as angle in relation to the parahippocampal gyrus (see Caciagli et al.^2019^). In a large study of 2008 participants (Cury et al. 2015), the presence of this anatomical feature was visible in 17% of left hippocampus and 6% of right hippocampus. Cury et al. (2020) replicated this result in PING, finding a similar incidence rate, as well as examined the genetic predictors. Incomplete hippocampal inversion has been associated with an increased risk of developing epilepsy (Gamss et al. 2009; Caciagli et al. 2019). Other features may be useful to examine in large datasets, but have not been yet, such as the presence of single or double cingulate sulci (Vogt et al. 1995; Cachia et al. 2016; Amiez et al. 2019) and orbitofrontal sulci patterns (Chiavaras and Petrides 2000; Nakamura et al. 2007; Li et al. 2019). Heschl’s gyrus morphology variants have been examined in one large dataset (Marie et al. 2016), but would benefit from further research.

### Robust Findings

**Fig. 5:**
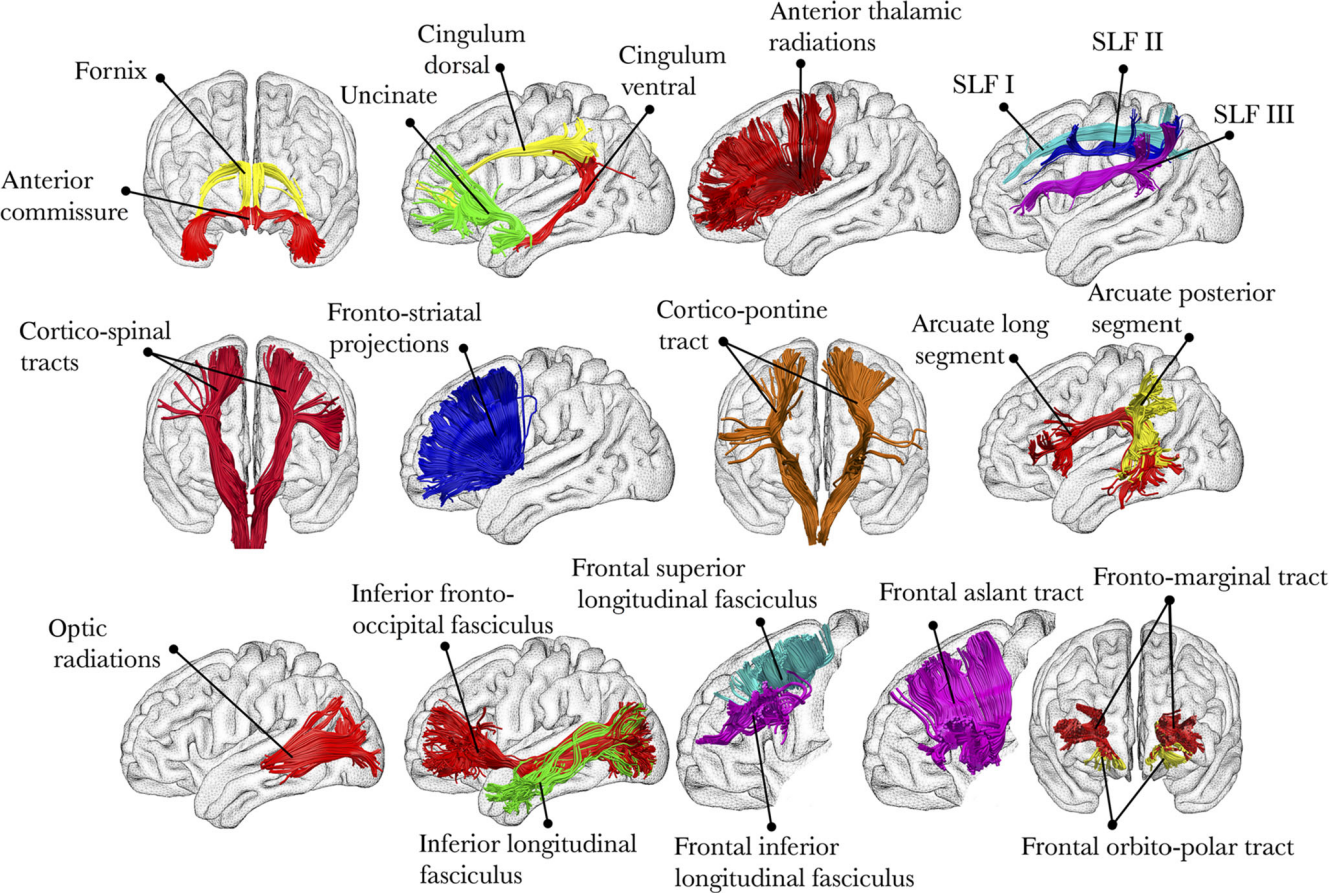
Overview of white-matter tracts. Reprinted from Thiebaut de Schotten et al. (2015)

Significant results based on large sample sizes are less likely to be due to random chance, and thus can be considered more robust (though admittedly can still occur due to systematic error) (Hung et al. 1997; Thiese et al. 2016; Madan 2016; Greenland 2019). Several studies have used the HCP task-fMRI data to evaluate the robustness of task condition contrasts and functional connectivity configurations (Barch et al. 2013; Shine et al. 2016; Schultz and Cole 2016; Shah et al. 2016; Westfall et al. 2017; Zuo et al. 2017; Nickerson 2018; Markett et al. 2020; Jiang et al. 2020). Margulies et al. (2016) used the HCP data to demonstrate gradients within default-mode network structure, providing significant insights into how sensory and association cortices communicate (Fig. 2a). Providing an example from a patient dataset, Cousineau et al. (2017) observed differences in white matter fascicles associated with Parkinson’s disease using the PPMI dataset, a result that was strengthened by the relatively large sample size of the dataset and the acquisition of test-retest DTI scans. See Fig. 5 for a summary of white-matter tracts.

**Fig. 6:**
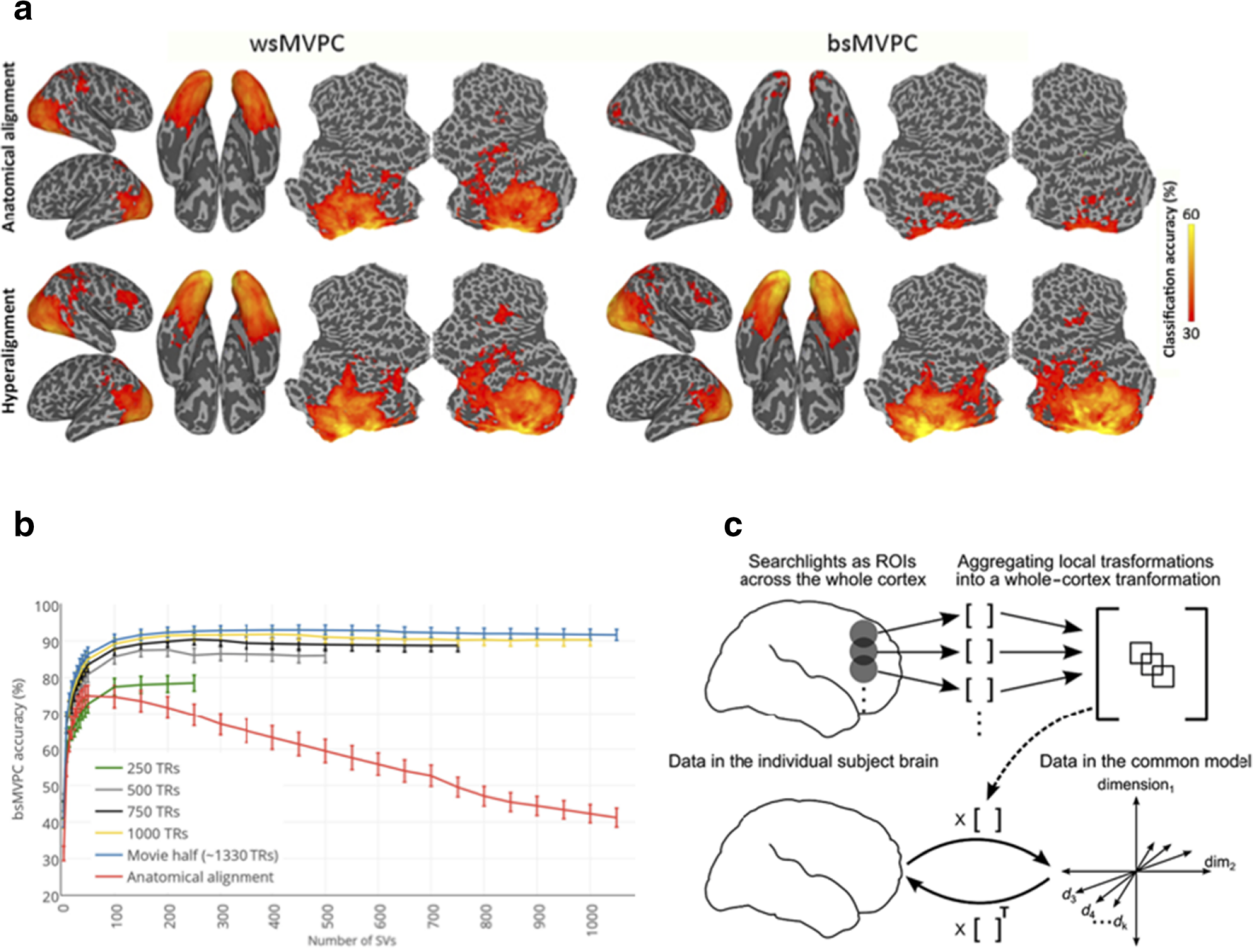
Performance of hyperalignment in comparison to conventional anatomical alignment. **a** Classification performance from a six-category animal localiser. wsMVPC denotes within-subject multivariate pattern classification; bsMVPC denotes between-subject. **b** Between-subject MVPC performance of movie-watching data, as a function of amount of data used in the hyperalignment. **c** Illustration of the method. Reprinted from Guntupalli et al. (2016)

As an increasing number of datasets collect movie watching data, this additionally affords the opportunity of bringing hyperalignment methods to the mainstream. Of course, movie watching/naturalistic stimuli themselves are able to provide insights into brain function in new ways that resting-state and task fMRI methods could not. That said, Haxby and colleagues have demonstrated that hyperalignment should be considered as an alternative to conventional anatomical-based normalisation. Briefly, conventional fMRI methods rely on than warping the structural MRI into a common space and then applying that non-linear transform to co-registered rest/task fMRI data. In contrast, hyperalignment uses the time-varying activations related to a movie-watching stimuli as a common, high-dimensional representation to serve as the transformation matrix to bring individuals into a common MRI space (Fig. 6c). A comparison of alignment methods is shown in Fig. 6a (task data from an six-category animal localiser shown). Despite comparable performance within-subject, conventional anatomical methods perform poorly across subjects. Analyses indicated that 10:25 min (250 TRs) of movie watching was sufficient for hyperalignment methods (Haxby et al. 2011; Guntupalli et al. 2016). More recent development of the connectivity hyperalignment method–as opposed to the original approach, now termed ‘response hyperalignment’–have improved the utility of the method in aligning connectivity data, but response hyperalignment nonetheless is advantageous in some situations (Guntupalli et al. 2018; Haxby et al. 2020).

### Reproducability

An added benefit of analysing public data is that outputs can be compared directly. With some datasets, this is the primary function of the dataset, such as with the 7 T test-retest (Gorgolewski et al. 2013) and NARPS datasets (Botvinik-Nezer et al. 2019; Botvinik-Nezer et al. 2020). With these datasets, researchers can test their ability to reproduce the analysis pipeline or develop new analysis pipelines and compare the output with previous results as benchmarks (i.e., analytical flexibility) (also see Silberzahn et al. 2018; Schweinsberg et al. 2021). This allows for confidence that the same input data was used, rather than an attempt to collect new data and replicate prior results. Some studies have taken this one step further, implementing a ‘multiverse’ approach in examining data through multiple methods, by the same research group (Carp (2012) and Pauli et al. (2016); also see Steegen et al. (2016) and Botvinik-Nezer et al. (2020)). In a similar vein, a recent large-scale collaboration used a subset of the HCP data to assess consistency across tractography segmentation protocols (Schilling et al. 2020). Here it was a clear benefit that public data that all researchers could access was already available (also see ADNI TADPOLE challenge: Marinescu et al. 2020).

**Fig. 7:**
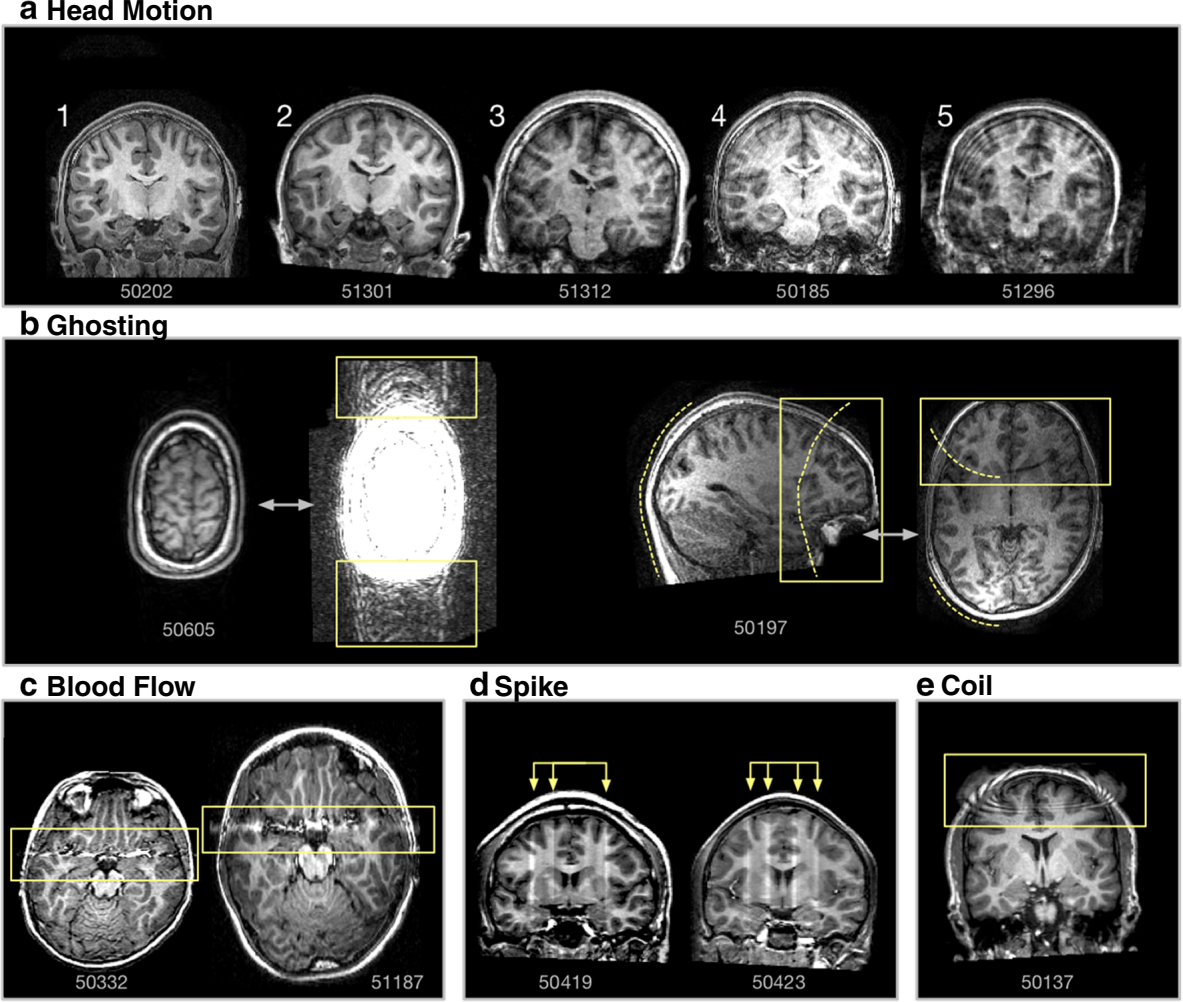
Examples of MRI artifacts in T1 volumes present in the ABIDE dataset. **a** Head motion artifacts, with increasing magnitude of motion left to right. Volumes comparable to images 1 and 2 would be suitable for further analysis, but those rated as 3 through 5 have too much head motion to be useable. While most of the ABIDE data is of reasonable quality, it is large dataset and includes participants with autism spectrum disorder as well as children, both factors known to be associated with increased head motion (Pardoe et al. 2016; Engelhardt et al. 2017; Greene et al. 2018). **b** Ghosting artifacts, visible as overlapping images. The example on the left is only visible in the background with a constrained intensity range, but still results in distortions in the image. The image on the right shows a clear duplicate contour of the back of the head. **c** Blood flow artifact, creating a horizontal band of distortion, here affecting temporal lobe imaging. **d** Spike noise artifact, resulting in inconsistent signal intensity. **e** Coil failure artifact, resulting in a regional distortion around the affected coil. Participant IDs are included below each image to allow for the further examination of the original 3D volumes. Artifact MRIs were identified with the aid of MRIQC (Esteban et al. 2017). Pre-computed results are available from https://mriqc.s3.amazonaws.com/abide/T1w_group.html

A related use is more teaching-oriented. Since the MRI data from these datasets are publicly available–at least after agreeing to the initial data-use terms. As such, they can readily be used as specific real-world examples of acquired data. To provide a concrete example of this, I made Fig. 7 to show instances of MRI artifacts using data from ABIDE. While this figure itself should be useful for those familiarising themselves with the neuroimaging data, I have included the participant IDs to allow interested readers to go one step further and examine the same MRI volumes that I used to make the figures.

**Fig. 8:**
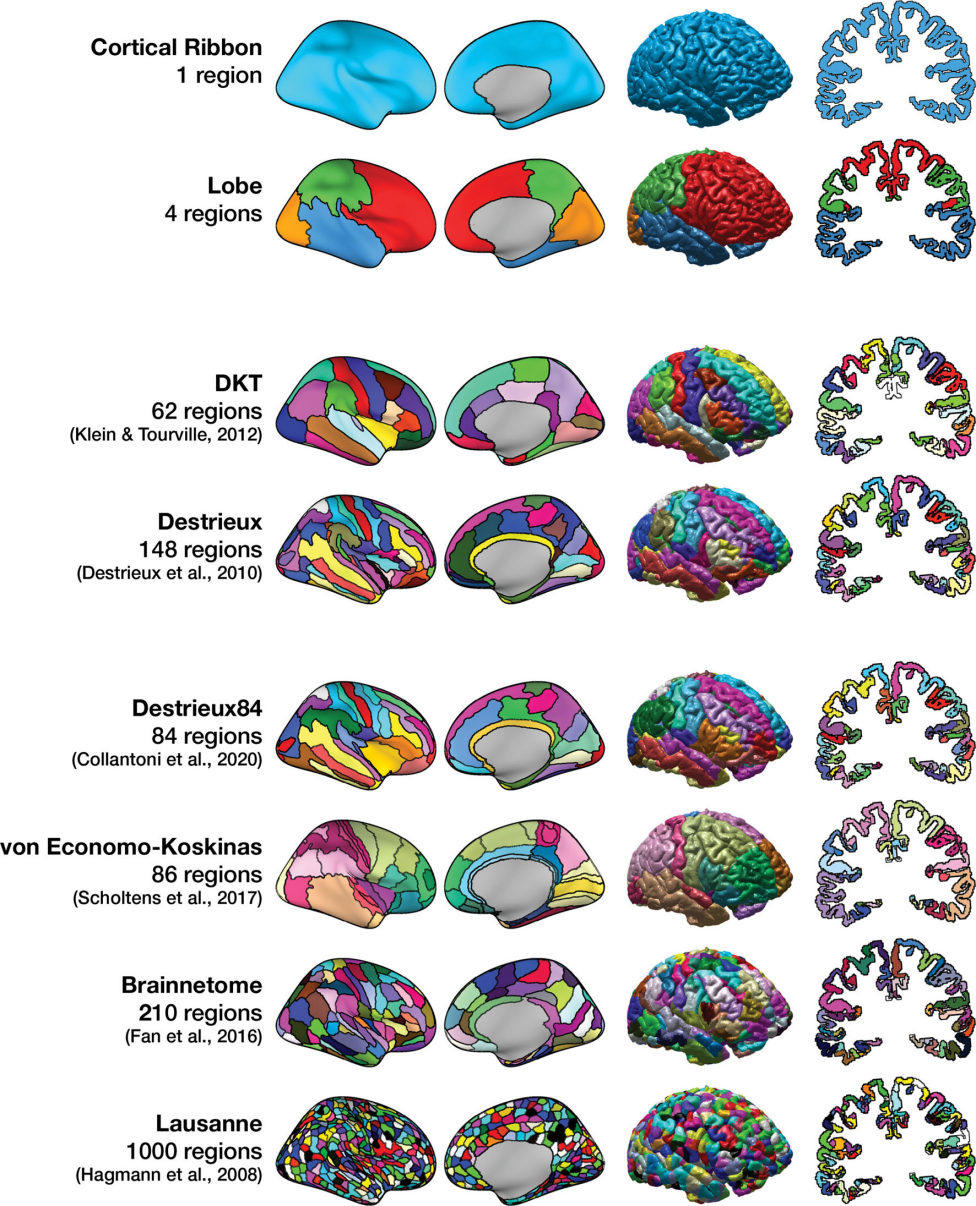
Overview of cortical parcellation approaches instantiated in FreeSurfer. Parcellations are shown on inflated and pial surfaces and an oblique coronal slice, reconstructed from an MRI of a young adult. Updated from Madan and Kensinger (2018) to include Collantoni et al. (2020) and more clearly show parcellation boundaries on the inflated surface; visualisations produced based on previously described methods (Madan and Kensinger 2016; Klein and Tourville 2012; Destrieux et al. 2010; Scholtens et al. 2018; Fan et al. 2016; Hagmann et al. 2008)

A further advantage of the large sample sizes available in many of the featured datasets is that they allow for cross-validation analyses, where analyses are conducted on subsets of the data and patterns of results can be evaluated as being replicable, particularly across multiple sites. Among other rigorous analyses, Abraham et al. (2017) use data from ABIDE and examine inter-site cross validation, where data is pooled across several sites and used to predict autism spectrum disorder diagnosis in other sites (also see Varoquaux 2018; Owens et al. 2019). Several age-prediction studies have similarly used several datasets to identify age-sensitive regions and predict age in independent datasets (e.g., Cole et al. 2015; Madan and Kensinger 2018; Bellantuono et al. 2021). This has been done with other topics as well, where large datasets such as HCP and ADNI are used as replication samples and works particularly well for studies that are otherwise examining individual differences (e.g., Hodgson et al. 2017; Madan & Kensinger, 2017a; Madan, 2019b; Richard et al. 2018; Young et al. 2018; Grady et al. 2020; Baranger et al. 2020; Baranger et al. 2020; Kharabian Masouleh et al. 2020; Weiss et al. 2020; Yang et al. 2020; van Eijk et al. 2020). See Fig. 8 for an overview of anatomical-based cortical parcellations.

**Fig. 9:**
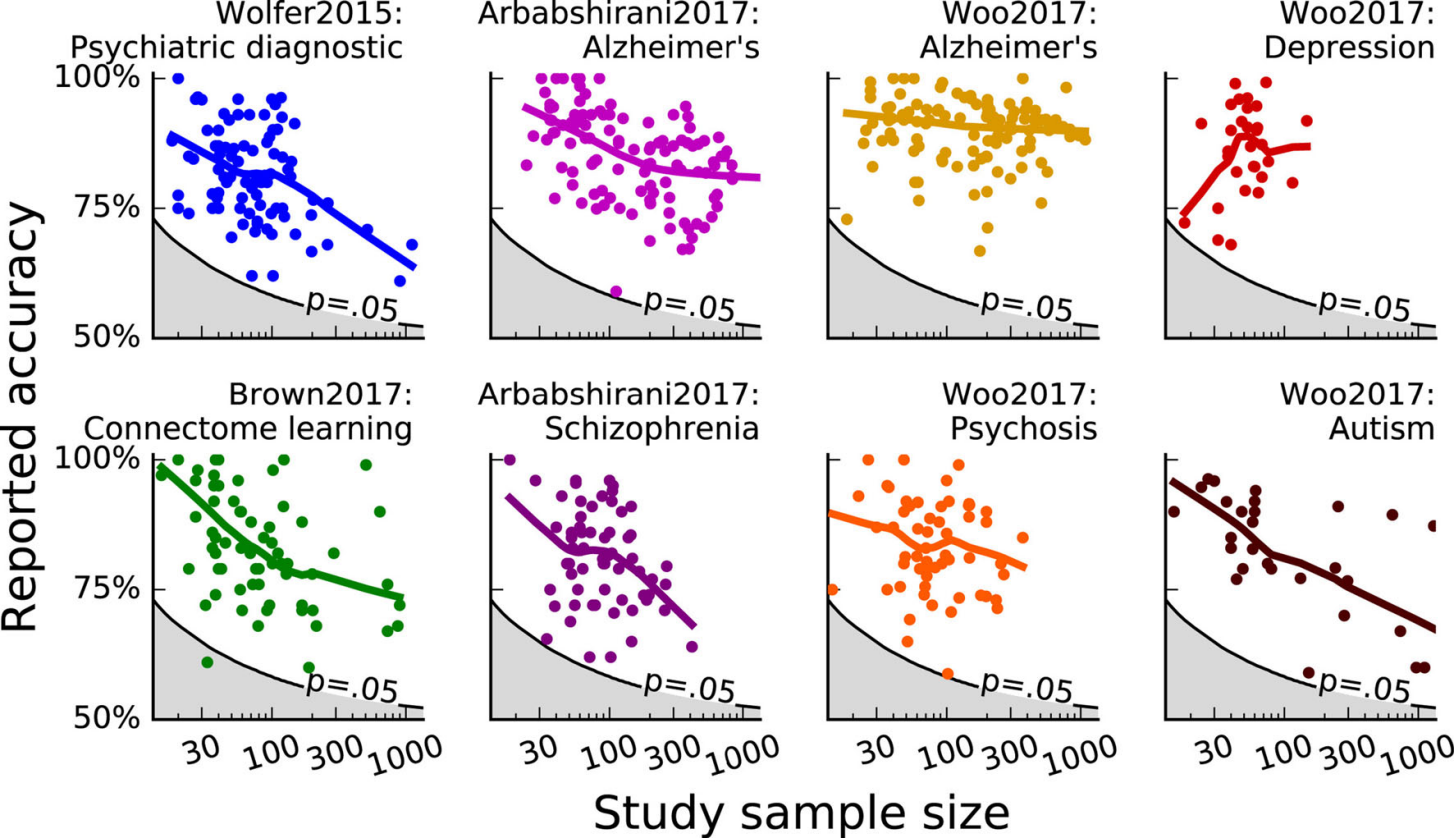
Reported prediction accuracy as a function of sample size for studies in different meta-analyses. Reprinted from Varoquaux (2018). Copyright 2018, Elsevier

Some studies have examined how results vary in relation to sample size, either in the form of a meta-analysis or through the analysis of subsets of the data, see Fig. 9 (Termenon et al. 2016; Varoquaux 2018; Zuo et al. 2019; Grady et al. 2020; also see Schönbrodt and Perugini 2013). It is well-established, unfortunately, that smaller cohorts often result in overestimation of effect sizes (Hullett and Levine 2003; Forstmeier and Schielzeth 2011; Varoquaux 2018). Larger datasets should result in more accurate effect sizes and, in principle, should yield more robust and generalisable findings. By necessity, larger datasets include more heterogeneous data than smaller datasets. Admittedly, the use of large datasets makes most analyses yield either clearly significant or non-significant results, due to the large sample sizes, thus making the distinction between the practical relevance or meaningful effect size important to consider (e.g., ‘smallest effect size of interest’; Lakens et al. 2018), rather than the statistical significance itself.

### Methods Development

Some uses of large open-access datasets have been for purposes that would not have been practical as the primary outcome of new data collection, but take advantage of existing data to refine analysis methods going forward. For example, Esteban et al. (2017) developed MRIQC to automatically and quantitatively assess MR image quality on a variety of metrics, using data from ABIDE and LA5c. Advanced Normalisation Tools (ANTs) (Tustison et al. 2014) is a volumetric pipeline for image registration, tissue segmentation, and cortical thickness estimation, among other structural MRI operations. This 2014 paper that demonstrating a rigourous evaluation of this comprehensive pipeline uses four open-access datasets (IXI, MMRR, NKI, OASIS1) to showcase the robustness of the pipeline, including example figures corresponding to specific individual MRI inputs. Davis (2021) recently used data from OASIS1 to examine variability in cortical depth (i.e., distance from the scalp to the cortical surface, through the skull) as a means of assessing regional variability for transcranial stimulation research. IXI and OASIS1 have been used as a training dataset for a large number of methodological developments, especially in relation to age-related effects (e.g., Schrouff et al. 2013; Yun et al. 2013; Romero et al. 2015; Auzias et al. 2015; Wang et al.^2016^). In another instance, Madan (2019a) developed a novel toolbox for quantifying sulcal morphology and evaluated the generalisability of the method across several healthy aging dataset–OASIS1 and DLBS–as well as SALD as a non-Western sample and CCBD to assess test-retest reliability. HCP, GSP, MASSIVE, and Maclaren et al. (2014) have also useful for assessing test-retest reliability (also see Madan and Kensinger2017b).

**Fig. 10:**
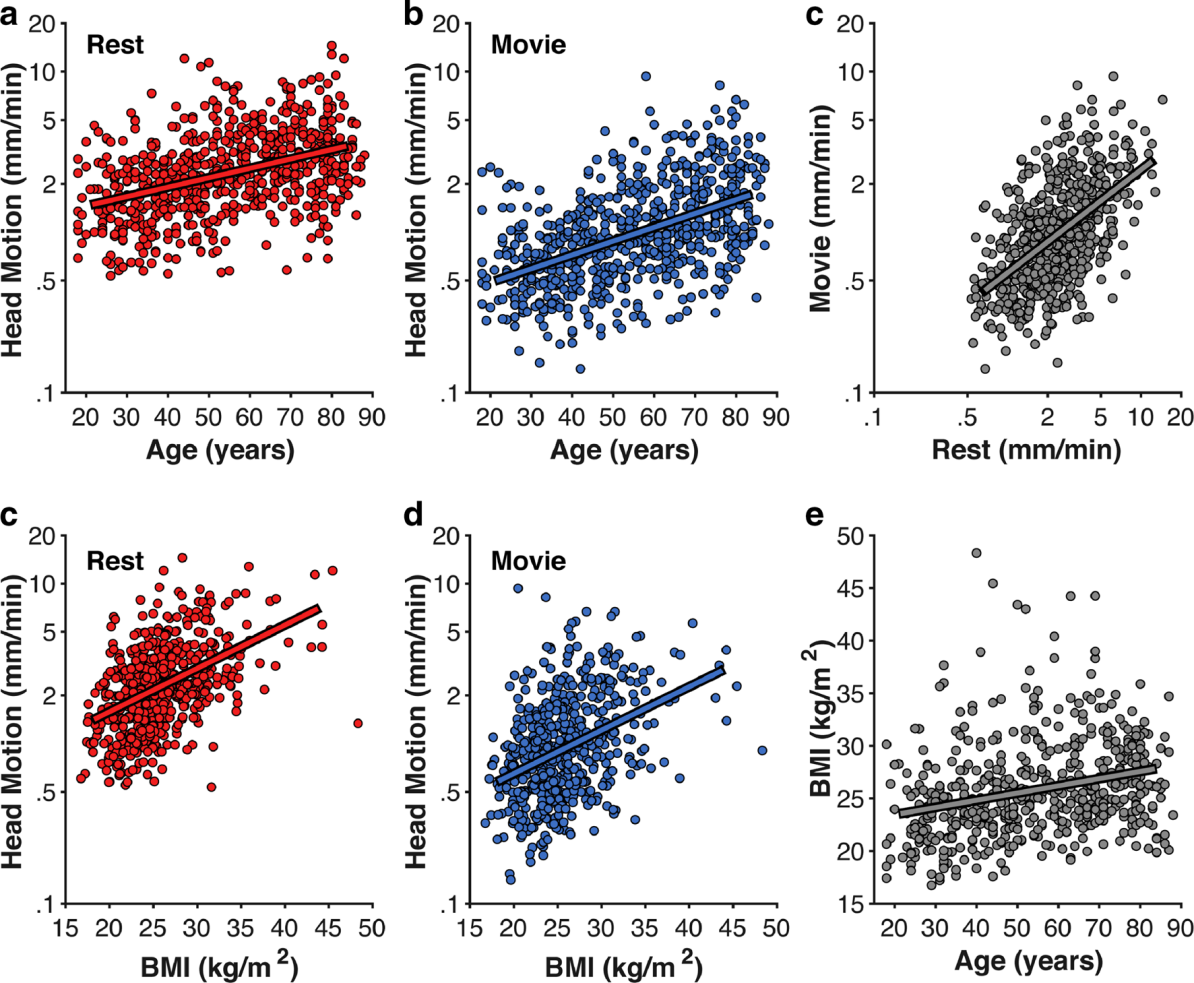
Correlations between head-motion during rest and movie-watching fMRI scans with age and body-mass index (BMI). Head motion axes are log-10 scaled to better show inter-individual variability. Reprinted from Madan (2018)

Madan (2018) used data from CamCAN to replicate a number of findings that have been previously shown, including increased head motion in older adults, decreases in head motion associated with movie watching, and weak but statistically significant effects of head motion on estimates of cortical morphology (Fig. 10). This lead to the proposal that watching a movie during the acquisition of a structural volume would improve data quality, though consideration is needed, e.g., this would be problematic if the structural volume was then followed by a resting-state sequence. Body–mass index (BMI) was also associated with increased respiratory-related apparent head motion, determined through the use of multiple estimates of head motion, and has since been supported by a further study that also used these large-scale datasets, Power et al. (2019). While the focus of Madan (2018) was aging effects on head motion, Power et al. (2019) examined head motion effects on fMRI signal, using the HCP, GSP, and MyConnectome datasets (complemented by additional within-lab datasets). Pardoe et al. (2016) have examined head motion effects across several clinical populations using data from ABIDE, ADHD-200, and COBRE; Zacà et al. (2018) examined head motion in the PPMI.

**Fig. 11:**
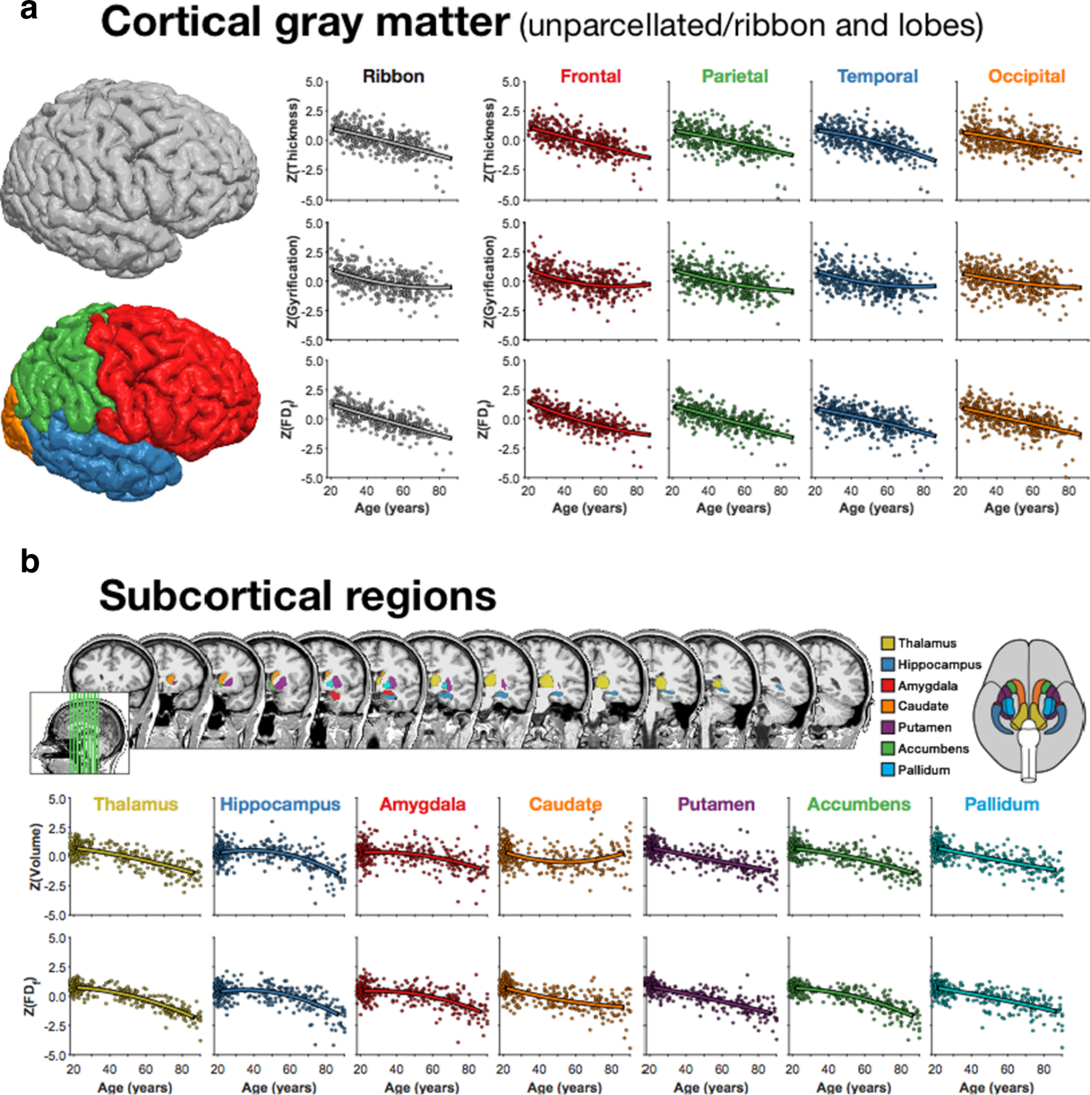
Age-related differences in brain morphology–characterised using cortical thickness, gyrification index, and fractal dimensionality–across the entire cortical gray matter (‘ribbon’) and for each lobe. Adapted from Madan and Kensinger (2016, 2017a)

Using data from ADNI, King et al. (2009, 2010) demonstrated that fractal dimensionality can be a more sensitive measure of brain structure differences associated with Alzheimer’s disease than conventional measures of cortical thickness and gyrification. Inspired by this work, Madan and Kensinger (2016) examined age-related differences in the IXI database and found this as well; later using IXI, OASIS1, and a within-lab sample to examine subcortical structure (Madan and Kensinger 2017a) (Fig. 11). Several later studies expanded on these initial findings (Madan and Kensinger 2017b, 2018; Madan 2018, 2019b, 2021), fully reliant on large open-access datasets.

## Closing Thoughts

Conducting a new neuroimaging study can easily have a budget upwards of 20,000 dollars (or pounds) for the MRI scan time, let alone the time and labour associated with participants and researchers involved. The value of already collected data grows greatly in the unlikely circumstances of a wide spread public health issue (as in our current COVID-19 pandemic), where contact between individuals must be minimised, but PhD training–as well as furthering our understanding of the brain–must continue.

In using these large-access open-access datasets, we must consider the decisions that went into the data we are now using. Some of these are still yet to be made, such as how to harmonise the MRI data from multiple sites or similarly how to reconcile potential differences in screening criteria between sites (particularly when data is aggregated at a later stage, rather than a planned multi-site study). Other decisions have already been made and simply need to be incorporated into the subsequent research despite limitations, such as the artifacts in the multiband sequence used in the HCP (Risk et al. 2018; McNabb et al. 2020) and specifics of the task design used in the ABCD study (Bissett et al. 2020). We also need to consider the prior use of the datasets, specifically to become over-reliant, and thus over-fit, our knowledge as a field to specific datasets. Given the current state of the field, this is of particular concern with HCP and ADNI–if too many analyses are based on these specific samples, that may bias our understanding of the brain. As an example, it is worth re-visiting the recruitment procedures for these studies and evaluating how representative they are of the desired population we may want to generalise or what sampling biases may be present, e.g., education level, socioeconomic status, response bias. For further, more focused discussions on current topics and using open-access neuroimaging datasets in specific contexts, please see the referenced papers: development (Gilmore 2016; Klapwijk et al. 2021), aging (Reagh and Yassa 2017), brain morphology (Madan 2017), naturalistic stimuli (Vanderwal et al. 2019; Finn et al. 2020; DuPre et al. 2020), head motion (Ai et al. 2021), non-human primates (Milham et al. 2020) data management (Borghi and Van Gulick, A. E. 2018), computational reproducibility (Kennedy et al. 2019; Poldrack et al. 2017; Poldrack et al. 2019; Carmon et al. 2020), and machine learning (Dwyer et al. 2018).

As a final set of remarks, I would like to direct readers to several articles to help deepen how they make think of the brain. Though we hopefully have sufficiently moved on from the days of circular inferences, Vul et al. (2009) remains an important article for those entering the field. Weston et al. (2019) raises many important considerations associated with working with secondary datasets, while Broman and Woo (2017) and Wilson et al. (2017) are essential reads for data organisation and scientific computing, respectively–both critical topics when working with large datasets. Pernet and Madan (2020) provides guidance for producing visualisations of MRI analyses. Eickhoff et al. (2018) and Uddin et al. (2019) provide insightful discussions for thinking about the structure of the brain.
